# Progress in research on the use of induced pluripotent stem cells in tumor prevention, therapy, and tumor modeling

**DOI:** 10.3389/fcell.2026.1968508

**Published:** 2026-10-16

**Authors:** Shaoshuai Liang, Hongyang Zhao, Ning Zuo, Bingqiang Zhang, Mengmeng Chen, Xiaoyan Zhang, Haoyun Zhang, Muhammad Omer Iqbal, Qi Wang, Dingcai Dong

**Affiliations:** 1 Life Valley (Qingdao) Health Technology Co., Ltd., Qingdao, Shandong, China; 2 Qingdao Engineering Research Center for Cellular Immunity and Early Cancer Screening, Qingdao Restore Biotechnology Co., Ltd., Qingdao, Shandong, China; 3 College of Biological Engineering, Qingdao University of Science and Technology, Qingdao, China; 4 Department of Pharmacology, School of Pharmacy, Qingdao University, Qingdao, China; 5 School of Basic Medical Science, Shandong Second Medical University, Weifang, China; 6 Neurologic Disorders and Regenerative Repair Lab of Shandong Higher Education, Shandong Second Medical University, Weifang, China

**Keywords:** cancer stem cells, induced pluripotent stem cells, prevention, therapy, tumor modeling

## Abstract

Induced pluripotent stem cell technology holds great promise for cancer research and immunotherapy. iPSCs possess pluripotency and unlimited self-renewal capacity, enabling their differentiation into various functional immune effector cells such as T cells, natural killer cells, and macrophages (Macs). Genetically engineered iPSC-derived immune cells have been shown to efficiently and specifically eliminate cancer cells in preclinical studies, and their safety and preliminary efficacy are currently being evaluated in early clinical trials. Although iPSC technology is a relatively recent development, it can be applied in a variety of ways to tackle biological challenges such as tumor heterogeneity, metastasis, and therapeutic resistance. Recent evidence has demonstrated the potential of iPSC-based tumor vaccines, which can leverage the shared transcriptomic characteristics and tumor-associated antigens of iPSCs and cancer cells, to activate CD8^+^ T cells, induce immune memory formation, and inhibit tumor growth. Moreover, iPSCs can be reprogrammed into cancer stem cell-like cells (CSC-like cells) by tumor microenvironmental cues or genetic mechanism, while cancer cells can be reprogrammed to cancer-derived iPSCs. These complementary approaches provide powerful platforms for modeling tumor initiation, investigating drug resistance mechanisms, and reversing malignant phenotypes. In this review, we discuss new applications of iPSCs in cancer immunotherapy, tumor vaccine development, and CSC-based disease modeling. The review concludes by examining some of the challenges facing the clinical translation of iPSCs, including tumorigenicity risks, low reprogramming efficiency, and the immunosuppressive tumor microenvironment. We also recommend possible solutions to these challenges, which can help to further advance the application of iPSCs in precision oncology and personalized cancer treatment.

## 1 Introduction

The global incidence of cancer is on the rise (GBD 2023 Cancer Collaborators, 2025); thus, the pace of developing translational medicine must be accelerated. Traditional treatment methods for cancer such as surgical resection, radiotherapy, and chemical drug treatment are currently the main intervention methods used in clinical practice. Although these methods show therapeutic effects in specific clinical scenarios, their efficacy is significantly limited owing to biological characteristics such as tumor metastatic potential and intratumoral heterogeneity (Allam et al., 2020; Wensink et al., 2021). This has led to the rapid development of tumor immunotherapy, tumor vaccines, and precise tumor models.

iPSC technology has emerged as a promising approach in cancer research owing to its unique properties. Similar to embryonic stem cells (ESCs), iPSCs regain pluripotency by introducing specific transcription factors into adult cells (Selvaraj, 2012). In 2006, Takahashi and Yamanaka first demonstrated that four transcription factors (*Oct4, Sox2, Klf4*, and *c-Myc* (OSKM)) restore pluripotency in mouse fibroblasts and generate iPSCs (Takahashi and Yamanaka, 2006). The same group established the human iPSC (hiPSC) system the following year (Takahashi et al., 2007). This breakthrough provides an ethically friendly and efficient alternative to controversial ESCs. Distinguished by their pluripotency, infinite proliferative capacity *in vitro*, and potential for directional differentiation into diverse functional cell types (Song et al., 2024; Mohite et al., 2024). iPSCs have emerged as a transformative platform bridging basic research and personalized clinical translation.

iPSC-derived immune cells represent a cancer treatment platform. Through directional differentiation, iPSCs produce a variety of immune cells, such as T cells, macrophages, NK cells, and other immune cells with anti-tumor activity (Xue et al., 2023; Zimmermannova et al., 2021). Among these, iPSC-derived T cells (iPSC-T) modified with chimeric antigen receptors (CAR) demonstrate strong anti-tumor effects *in vitro* and *in vivo*, exhibiting superior expansion, persistence, and cytotoxicity compared to traditional CAR-T cells (Mishra and Kalyuzhny, 2024; Guo et al., 2026). iPSC-derived NK (iPSC-NK) cells attract considerable attention due to their mature phenotype, strong cytotoxicity, and scalability. Gene editing can further enhance their cytokine release, expansion efficiency, and tumor-targeted killing (Goldenson et al., 2022). iPSC-derived macrophages (iPSC-Macs, iMacs) offer a new solid tumor immunotherapy strategy based on phagocytosis and cytokine secretion (Shah et al., 2024). The iPSC platform enables genetic modification by regulating immune signaling pathways and enhancing anti-tumor function (Xue et al., 2023; Zimmermannova et al., 2021). With the optimization of differentiation protocols and progress in gene editing technology, iPSC-derived immune cells will improve the standardization, generalization, and efficiency of cancer immunotherapy.

Beyond iPSC-derived immune cell therapies, iPSCs can be further engineered into tumor vaccines that activate systemic anti-tumor immunity. iPSC vaccines show breakthrough potential in cancer prevention, achieving tumor removal and long-term remission in various malignant tumor models by simultaneously activating cellular and humoral immune responses. Considering that previous traditional vaccines cannot overcome the lack of antigenicity, the new method can stimulate the activation of specific new antigen-reactive T cell responses by introducing an antigen-engineered autologous iPSC vaccine strategy, promoting tumor regression, and reducing the risk of distant metastasis of tumors through local radiotherapy (Huang et al., 2024). Additionally, iPSC-derived exosome vaccines circumvent the tumorigenic risk while retaining a broad repertoire of stem cell-associated antigens, providing new approaches to the design of safe and effective broad-spectrum vaccines (Wang et al., 2023). Despite the immunosuppressive microenvironment, the multi-antigen presenting capability of iPSC vaccines renders them ideal for preventing the development of heterogeneous tumors (Jwo et al., 2025).

Normal iPSCs can be converted into CSC-like cells using tumor microenvironment (TME) components or via genetic manipulation. Mouse iPSCs (miPSCs) can be transformed into CSCs *in vivo* upon co-transplantation with pancreatic cancer cells. Converted iPSCs exhibit markers and functional characteristics typical of CSCs (Hassan et al., 2022). Secretory components of the TME are key for converting iPSCs into CSCs (Abu Quora et al., 2021). Tumor-derived extracellular vesicles (TDEVs) drive iPSC-to-CSC conversion by delivering specific molecular cargo. Introducing oncogenes or knocking out tumor suppressors in iPSCs increases microenvironment sensitivity. Moreover, culturing iPSCs under specific mutation and tumor conditions may readily induce CSC-like characteristics (Sarker et al., 2024). Cancer mutations provide the initial transformation motivation, while TME components maintain CSC malignancy (Shamsian et al., 2022; Rodrigues et al., 2022; Giron-Michel et al., 2025). This deeper understanding of cancer initiation highlights directions for developing individualized iPSC-based therapies. Beyond converting normal iPSCs into CSCs, iPSC technology also offers a powerful platform for reversing the malignant phenotype of cancer cells. Specifically, iPSC technology can reprogram cancer cells to pluripotency, facilitating the establishment of highly plastic tumor models. The transcription factor networks and epigenetic regulation are highly similar, which enables the production of cancer-derived iPSCs (cancer-iPSCs) by reprogramming the cancer cells (Klein and Knoepfler, 2021). Although cancer cells harbor irreversible genomic changes, their malignant phenotypes can be significantly inhibited by reprogramming them into pluripotent stem cells and then differentiating them (Guo et al., 2025; Shamsian et al., 2022). CRISPR-Cas9 editing of iPSCs helps simulate progressive mutation accumulation during cancer development, modeling human cancer evolution (Wang et al., 2021). iPSC technology provides innovative tools for cancer research, spanning malignant phenotype reversal, disease modeling, and treatment development.

This article first discusses applications of iPSC-derived immune cells and tumor vaccines in cancer immunotherapy. Next, the bidirectional change between iPSCs transforming into cancer stem-like cells and cancer cells transforming into cancer-iPSCs is discussed. Furthermore, iPSC-derived tumor models outperform traditional organoids in scalability and patient specificity for drug screening, offering a more reliable platform for precise anticancer therapy; however, model standardization and safety remain challenges. In summary, iPSC technology advances the standardization, engineering, and personalization of tumor immunotherapy. Despite some challenges, integrating gene editing and three-dimensional culture systems positions the iPSC platform well to provide breakthrough cancer treatments.

## 2 iPSC-derived immune cells

iPSCs can be generated using somatic cell reprogramming technology. The stability and standardization of the technology have ensured that iPSCs can be expanded and differentiated into various functional immune effector cells for tumor immunotherapy. This technology can support precise gene editing, laying the foundation for the development of “off-the-shelf” cell products (Mishra and Kalyuzhny, 2024; Basilio-Queiros et al., 2025). iPSCs can be generated via the reprogramming of somatic cells of patients or healthy donors and can then be differentiated into immune cells with specific functions (as shown in Figure 1). This iPSC-based strategy can overcome the limitations of traditional autologous cell therapy, such as limited cell sources and prolonged preparation cycles, while enhancing treatment accessibility and consistency via standardized production (Shah et al., 2024; Netsrithong et al., 2023).

**FIGURE 1:**
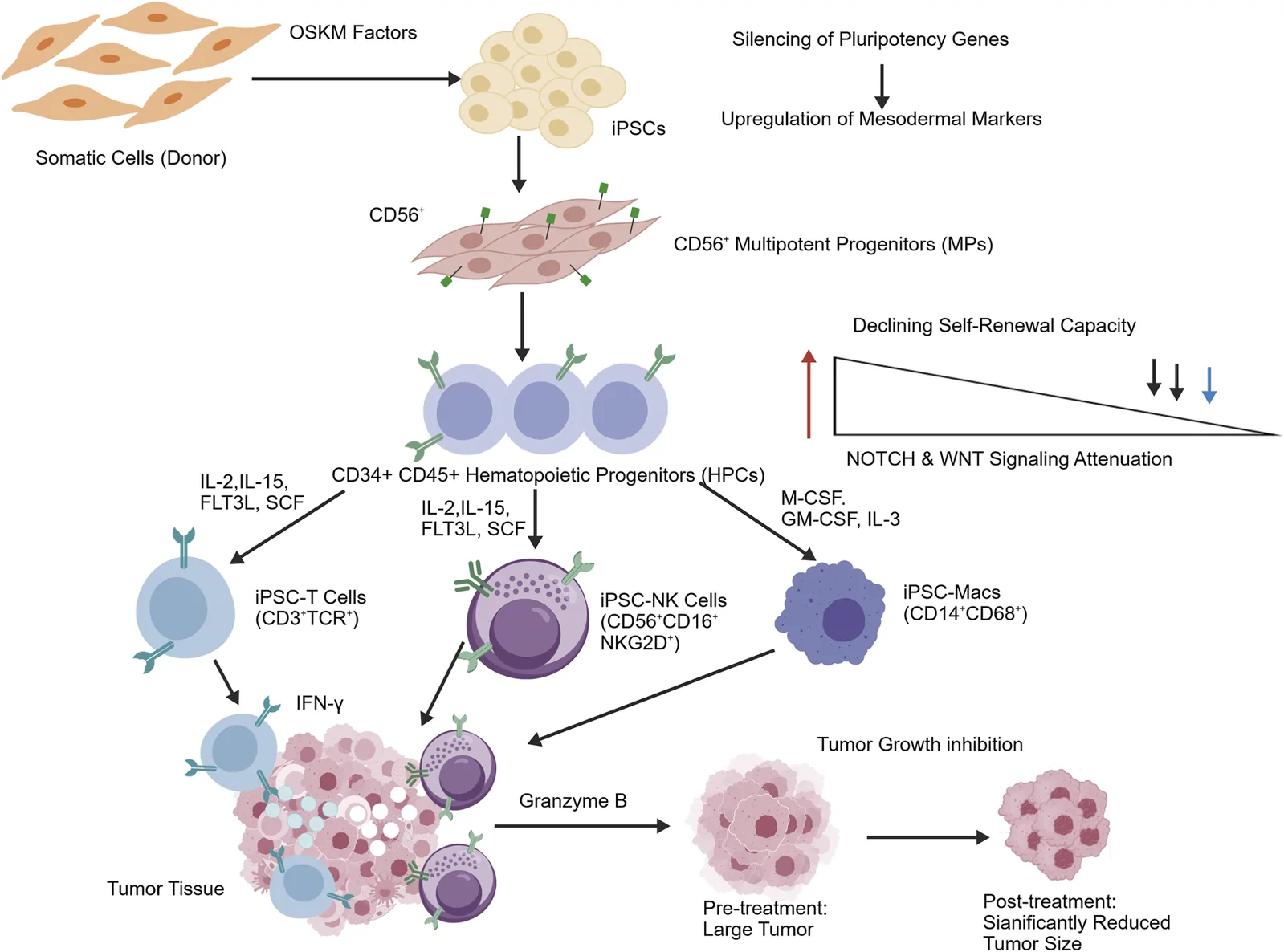
Overview of iPSC-derived immune cells. Complete mechanism of iPSC generation via somatic cell reprogramming and the multi-step directed differentiation pathways towards immune cells for cancer immunotherapy applications. Donor somatic cells are reprogrammed into iPSCs via OSKM factors; subsequently, iPSCs undergo mesodermal induction to generate CD56^+^ mesodermal progenitor cells (MPs). The intermediate MP population further differentiates into CD34^+^ CD45^+^ hematopoietic progenitor cells (HPCs), accompanied by a progressive decline in self-renewal capacity. Ultimately, under the regulation of specific cytokine microenvironments, HPCs undergo directed differentiation into functionally mature iPSC-derived immune effector cells, such as T cells, NK cells, and macrophages. iPSC-NK and iPSC-T cells infiltrate tumor tissues, releasing IFN-γ and granzyme B to significantly suppress tumor growth.

### 2.1 iPSC-derived T cells

The emergence of iPSC technology has provided a solution to the problem of cytotoxic T lymphocyte depletion in tumor immunotherapy. iPSC-T cells represent a strategic next-generation immunotherapy focus, providing an off-the-shelf, infinitely amplifiable, and readily editable cell source. By reprogramming antigen-specific T cells into T cell-induced pluripotent stem cells (T-iPSCs), the original T cell receptor (TCR) information can be retained, and the cells can be differentiated into T-iPS-T cells with the same TCR specificity (Ishiguro et al., 2023; Flippe et al., 2022). To enhance differentiation efficiency, Zhao et al. established a TCF7-RUNX1 fluorescence reporter in iPSC lines. Using endogenous gene labeling, they tracked iPSC-to-T cell differentiation in real time, identifying the RUNX1^high^/TCF7^mid-stage^ as a critical window for generating functional CD8 αβ T cells (Zhao et al., 2024). For functional optimization, CRISPR editing targeting the *RAG1/RAG2* recombinase genes in T-iPSCs effectively blocks endogenous random TCR rearrangement (Minagawa et al., 2018). This strategy produces antigen-specific CD8αβ T cells with a single stable TCR, significantly improving TCR repertoire homogeneity (Wu et al., 2023; Gardner et al., 2021).

Combining epigenetic reprogramming with a matrix-free culture system yields EZ-T cells with highly diverse TCR libraries, molecularly resembling peripheral blood TCR αβ T cells. Upon activation, they produce effector molecules and exhibit anti-tumor activity (Jing et al., 2025). The iPSCs obtained from CD62L^+^ naïve T cell reprogramming can be reshaped into functional αβ CAR T cells through the synergistic effects of CAR gene programming and an *in vitro* differentiation system (Wang et al., 2022). Trimodal T cells constructed by synchronous introduction of CAR, TCR, and humanized CD16 (hnCD16) provide an innovative methodology to overcome tumor heterogeneity (Yang B. H. et al., 2025). For treating solid tumors, Yang et al. developed trimodal iPSC-T cells that simultaneously express CAR, TCR, and hnCD16, which were designed to achieve the synergistic removal of heterogeneous tumor groups with antibody-dependent cell-mediated cytotoxicity (ADCC) (Yang B. H. et al., 2025). Udea. et al., 2023 Achieved anti-tumor activity comparable to that of primary CAR T cells by screening IL-15/IL-15Rα co-expressing clones, enabling engineered hiPSC-derived CAR T cells to exhibit therapeutic efficacy in multiple tumor-bearing animal models (Ueda et al., 2023). Compared with traditional methods, iPSC-T cells exhibit superior proliferation, persistence, and antitumor activity, with standardized platforms resolving batch variability and lengthy production cycles in autologous therapies (Mishra and Kalyuzhny, 2024; Netsrithong et al., 2023).

From the translational standpoint, iPSC-T cells have progressed from basic proof-of-principle experiments to preliminary clinical trials, demonstrating the feasibility of standardized off-the-shelf allogeneic therapies (Chen et al., 2025; Fang et al., 2025; Mishra and Kalyuzhny, 2024). FT819, an iPSC-derived CD19 CAR-T therapy in which the CD19 1XX CAR is knocked into the TRAC locus while ablating the endogenous TCR, has shown promising tumor-killing efficacy against B-cell malignancies and effective B-cell depletion in patients with systemic lupus erythematosus in a phase I trial, without causing any dose-limiting toxicities, immune effector cell-associated neurotoxicity syndrome, or graft-versus-host disease, and with only mild cytokine release syndrome (Fang et al., 2025). Simultaneously, FT825/ONC 2025, an iPSC-derived HER2-targeting CAR-T product, is being evaluated in a phase I trial for the treatment of HER2-positive advanced solid tumors (Fang et al., 2025). However, iPSC- T cell-based therapeutics mostly remain in the phase I stage of dose escalation trials, and challenges in differentiation efficiency, long-term persistence, tumorigenicity, and good manufacturing practice-compliant production must be resolved before registration trials become feasible (Fang et al., 2025; Chen et al., 2025). It should also be emphasized that the available clinical evidence for iPSC-T cell products is limited to early-phase, non-randomized studies with small patient numbers; the durability of clinical responses and the long-term safety of these products therefore require confirmation in larger, controlled trials (Fang et al., 2025; Chen et al., 2025).

### 2.2 iPSC-derived NK cells

NK cells, key effector cells of innate immunity, directly lyse tumor- or virus-infected target cells and coordinate immune responses via cytokine secretion (Vivier et al., 2008; Bottino et al., 2024). However, large-scale acquisition of highly purified NK cells from peripheral or umbilical cord blood is limited by donor scarcity and expansion bottlenecks. iPSC-directed differentiation of iPSCs into NK cells offers a new immunotherapy paradigm. iPSC-NK cells possess unique advantages: they can be obtained through a feeder-free differentiation system (Kiran et al., 2024), and the NK cells generated from a clonal starting cell population are highly homogeneous (Goldenson et al., 2022), overcoming donor variability. Li et al. (2018) generated CAR-expressing NK cells from iPSCs (NK-CAR-iPSC-NK) and enhanced cancer cell cytolytic activity through CAR-mediated targeting (Li et al., 2018). iPSC-NK cells can be improved using gene-editing technology. Cai et al. established CD226-overexpressing iPSC-NK cells showing typical phenotypes, enhanced anti-acute myeloid leukemia (AML) activity, increased cytokine release at low effector-to-target ratios, and upregulated cytotoxicity/immune activation pathways (Cai et al., 2025). This overexpression reduced tumor burden, prolonged survival, and alleviated systemic inflammation in AML models. Targeting and cytotoxicity can be enhanced by removing checkpoints or engineering CARs. Qin et al. knocked out the immune checkpoint protein NKG2A expressed by NK cells in human pluripotent stem cells and differentiated them into iPSC-NK cells. The NKG2A knockout significantly enhanced the killing ability of these cells against tumors and viral infections (Qin et al., 2024). CALHM2-knockout CAR NK cells exhibit stronger *in vivo* antitumor effects and overcome resistance (Peng et al., 2025). Wang et al. further engineered CD70-targeted CAR-iNK cells (70CAR-iNK) against hematological and solid tumors (Wang L. et al., 2025). Functionally, iPSC-NK cells exhibit mature phenotypes and potent cytolytic activity (Lin et al., 2023). The cytotoxic effect of NK cells on autologous or allogeneic iPSC-derived cells is not restricted by the HLA-I expression status of the targets. Indeed, target cells lacking HLA-I are readily killed via the “missing-self” recognition mechanism, and this cytotoxicity is further augmented by a concomitant reduction in HLA-I and upregulation of ligands for activating NK-cell receptors (Bogomiakova et al., 2023). iPSCs differentiate into phenotypically mature, highly cytotoxic NK cells, providing homogeneous populations for cancer immunotherapy (Lin et al., 2023; Kiran et al., 2024; Tang et al., 2021).

Notably, this preclinical progress was quickly followed by clinical transition. Specifically, iPSC-NK cells have been tested in phase 1 clinical trials, which provides important evidence of the feasibility, safety and efficacy of the use of iPSC-derived cells for clinical purposes. In a landmark first-in-human trial, Ghobadi et al. evaluated iPSC-derived CD19-directed CAR-NK cells in patients with relapsed or refractory B-cell lymphoma, demonstrating that iPSC-derived CD19-directed CAR-NK cells can be safely used in the clinic (Ghobadi et al., 2025). In this trial, 86 patients were treated. The maximum tolerated dose was not reached, cytokine release syndrome occurred in 6% (regimen A) and 13% (regimen B) of patients (maximum grade 2) without neurotoxicity, and deep and durable responses were observed in those with both indolent and aggressive lymphomas (Ghobadi et al., 2025). Additionally, Strati et al. conducted a multicentre, open-label phase 1 study of off-the-shelf iPSC-NK cell therapy (FT516) in relapsed or refractory B-cell lymphoma, further establishing the safety and feasibility of CD16-engineered iPSC-NK cell products in the allogeneic setting (Strati et al., 2025). Among the 55 patients treated, no dose-limiting toxicities were reported, and objective responses were achieved in 32 (58%) patients, although grade ≥3 neutropenia was common (84%) (Strati et al., 2025). Collectively, these phase 1 trials represent important milestones, affirming that iPSC-NK cells can be manufactured at clinical scale, administered safely, and elicit measurable anti-tumor activity, thereby validating the transition of iPSC-NK cell therapies from bench to bedside. Nevertheless, durable responses are currently achieved in only a subset of patients, and the long-term efficacy and persistence of these products remain to be established in larger clinical studies. Key barriers to overcome include limited *in vivo* persistence and expansion of the transferred cells, immunosuppressive tumor microenvironments, and potential loss of target antigens (Myers and Miller, 2021; Laskowski et al., 2022).

### 2.3 iPSC-derived Macs

Macrophages constitute the largest immune cell population in the solid TME (Li D. et al., 2024), with infiltration stemming from bidirectional interactions with cancer cells and the interstitial microenvironment. Their phenotypes and functions are highly heterogeneous, with subgroups playing antagonistic roles in tumor processes (Nalio Ramos et al., 2022). Macrophages perform functions including chemotaxis, tumor site infiltration, and antigen cross-presentation. CAR-iMacs targeting prostate stem cell antigens significantly amplify anti-tumor lethality via ADCC and coordinated pro-inflammatory cytokines release (Shah et al., 2024; Qian and Liu, 2025). iMacs efficiently and reproducibly produce macrophages exhibiting typical M1/M2 polarization, phagocytosis, and inflammatory factor regulation, widely used in disease models and immune regulation studies (Lyadova and Vasiliev, 2022; Ackermann et al., 2024). Density-dependent phenotypic regulation unique to iPSCs alters cell morphology and significantly affects inflammatory factor and chemokine secretion patterns in resting or activated states, which has been verified in THP-1 cells, primary monocytes, and induced microglia (Vaughan-Jackson et al., 2022). The genetic engineerability, innate tumor infiltration capacity, and unlimited expansion potential of iPSC-Macs as a progenitor cell source have enabled new research and application avenues in cancer immunotherapy.

In contrast to iPSC-T cells and iPSC-NK cells, iPSC-Macs are relatively less advanced with research being primarily at a preclinical stage with no clinical trials conducted as yet (Lindenbergh and van der Stegen, 2025). Developing upon the first-generation CD3ζ-CAR macrophages, second generation CAR-iMac cells expressing a CD3ζ–TIR signaling domain for orthogonal phagocytosis and antigen-dependent M1 polarization showed significantly improved anti-tumor efficacy against solid tumors (Lei et al., 2024). Toward clinical translation, allogeneic CAR-iMacs targeting prostate stem cell antigen (PSCA), co-engineered with membrane-bound interleukin 15 (IL-15) and a truncated epidermal growth factor receptor (EGFR) suicide switch, have demonstrated potent antitumor activity against pancreatic cancer and can be cryopreserved as an off-the-shelf product; however, their evaluation remains confined to preclinical models (Shah et al., 2024). iPSC-Macs, despite promising preclinical advances, remain to be validated in first-in-human studies (Zhu et al., 2026). Overcoming barriers such as CD47-SIRPα-mediated evasion of phagocytosis (Matlung et al., 2017) and immunosuppressive TME-driven M2 polarization (Yang Y. et al., 2025) will therefore be critical for translating iPSC-Macs into effective cancer therapies.

### 2.4 Other iPSC-derived immune cells

iPSC differentiation technology now includes generating various immune cells beyond T cells, such as NK cells, macrophages, dendritic cells (DCs) (Oba et al., 2021), invariant natural killer T cells (Yamada et al., 2016; Kitayama et al., 2016), and myelomonocytic cells (Imamura et al., 2018), providing diverse sources for cancer immunotherapy. CAR-modified iPSC-derived neutrophils have been shown to exhibit superior and specific cytotoxicity against tumor cells through direct contact and reactive oxygen species generation (Chang et al., 2022). iPSC-DCs enhance T cell activation through CD40^−^CD40L-mediated crosstalk with T cells, or upon CD40 engagement by T cell-expressed CD40L (Xue et al., 2023; Wang et al., 2024). Researchers are actively investigating the conversion of pluripotent stem cells into γδ T cells or cytokine-induced killer cells. Watanabe et al. generated γδ T cell-derived iPSCs (γδT-iPSCs) harboring rearranged TCRG and TCRD loci from zoledronate/IL-2-stimulated whole peripheral blood mononuclear cell (PBMC) cultures; these γδT-iPSCs could be redifferentiated into CD34^+^CD43^+^ hematopoietic progenitors, establishing a renewable platform for major histocompatibility complex (MHC)-unrestricted γδ T cell-based immunotherapy (Watanabe et al., 2018). These cells combine the toxic effects of NK-like cells and memory properties of T cells, providing a new option for tumor immunotherapy (Zimmermannova et al., 2021). Notably, iPSC-derived invariant natural killer T cells (iNKT) exhibit NK-like direct cytotoxicity and, upon activation by α-galactosylceramide-pulsed antigen-presenting cells, can effectively induce cancer-reactive memory T-cell responses, further highlighting the therapeutic versatility of iPSC-derived immune cells (Aoki et al., 2026). Clinically, in a phase 1 first-in-human trial, allogeneic iPSC-derived iNKT cells administered intra-arterially to ten patients with recurrent head and neck squamous cell carcinoma were well tolerated, with only one dose-limiting toxicity (grade 3 skin rash) and no other serious adverse events. Tumor progression was suppressed in two patients, who exhibited clonal expansion of memory- and effector-phenotype CD8^+^ T cells and activation of the IFN-γ signaling pathway, providing the first clinical evidence of the antitumor potential of iPSC-derived iNKT cells in solid tumors (Iinuma et al., 2025). These advancements broaden iPSC-derived immune cell applications and enhance treatment efficiency through multitarget synergy, supporting off-the-shelf immunotherapy development.

## 3 iPSC-derived tumor vaccines

iPSCs share transcriptomic and cellular characteristics with cancer cells and express a broad spectrum of tumor-associated antigens (Zhai et al., 2024; Qiao et al., 2025; Ouyang et al., 2021; Jwo et al., 2025). This shared antigenic feature positions iPSCs as a promising platform for broad-spectrum cancer vaccines. The idea of immunizing animals with fetal tissue to prevent the growth of transplantable tumors was first proposed more than a century ago (Brewer et al., 2009). iPSCs and ESCs are highly similar, with consistent expression of core pluripotency proteins (Brenes et al., 2024; Pei et al., 2023) and carcinoembryonic antigens (Ghosh et al., 2011). Li et al. (2009) found that undifferentiated human ESCs induce potent, specific, and durable anti-tumor immunity in immunocompetent mice, inhibiting CT26 colon cancer growth and prolonging survival (Li et al., 2009). Zhang et al. (2012) showed that human ESC-based vaccines safely elicit anti-ovarian cancer immunity and block metastasis in xenogeneic rats (Zhang et al., 2012). iPSC-based tumor vaccines offer advantages over ESCs by avoiding ethical controversies and source limitations; their autologous nature reduces the immune rejection risk in transplantation models (Jin et al., 2023; Zhong et al., 2022). When combined with CpG adjuvant, iPSC-based vaccines simultaneously stimulate cytotoxic antitumor CD8^+^ T cell effector and memory responses and induce cancer-specific humoral immune responses, while relieving immunosuppression by reducing immunosuppressive CD4^+^ Tregs. These vaccines prevent tumor formation in multiple murine models (Jwo et al., 2025; Kooreman et al., 2018; Ouyang et al., 2021; Krishnan et al., 2025).

Conventional iPSC-based cancer vaccines exhibit limited *in vivo* efficacy due to low immunogenicity and susceptibility to the immunosuppressive TME. Various strategies have been developed to enhance vaccine efficacy (Figure 2). Inserting neoantigen epitopes into iPSCs via genetic engineering can enhance antigen-specific T cell responses (Huang et al., 2024). In mouse models of triple-negative breast cancer, neoantigen-engineered autologous iPSC vaccines showed stronger cytotoxicity and effectively eliminated cancer cells. Combined with radiotherapy, this approach reduces distant metastasis risk. iPSC membranes, rich in tumor-associated antigens highly expressed in cancer cells but low in normal tissues, can encapsulate adjuvant nanoparticles to form vaccines that stimulate broad-spectrum anti-tumor immunity, effective across five mouse tumor models (Li et al., 2025). Zhai et al. (2021) utilized modified lipid materials to expand red blood cell membranes into coalesced nanocarriers, preserving membrane characteristics while increasing drug-loading capacity. Encapsulating iPSC proteins into iPSC@RBC-Mlipo nanovaccines promoted spleen cell accumulation, systemic anti-tumor immune activation, and metastasis inhibition in mice. This delivery system, based on antigens wrapped in damaged red blood cell membranes, can be specifically captured by the spleen, significantly enhancing antigen-presenting efficiency and vaccine efficacy.

**FIGURE 2:**
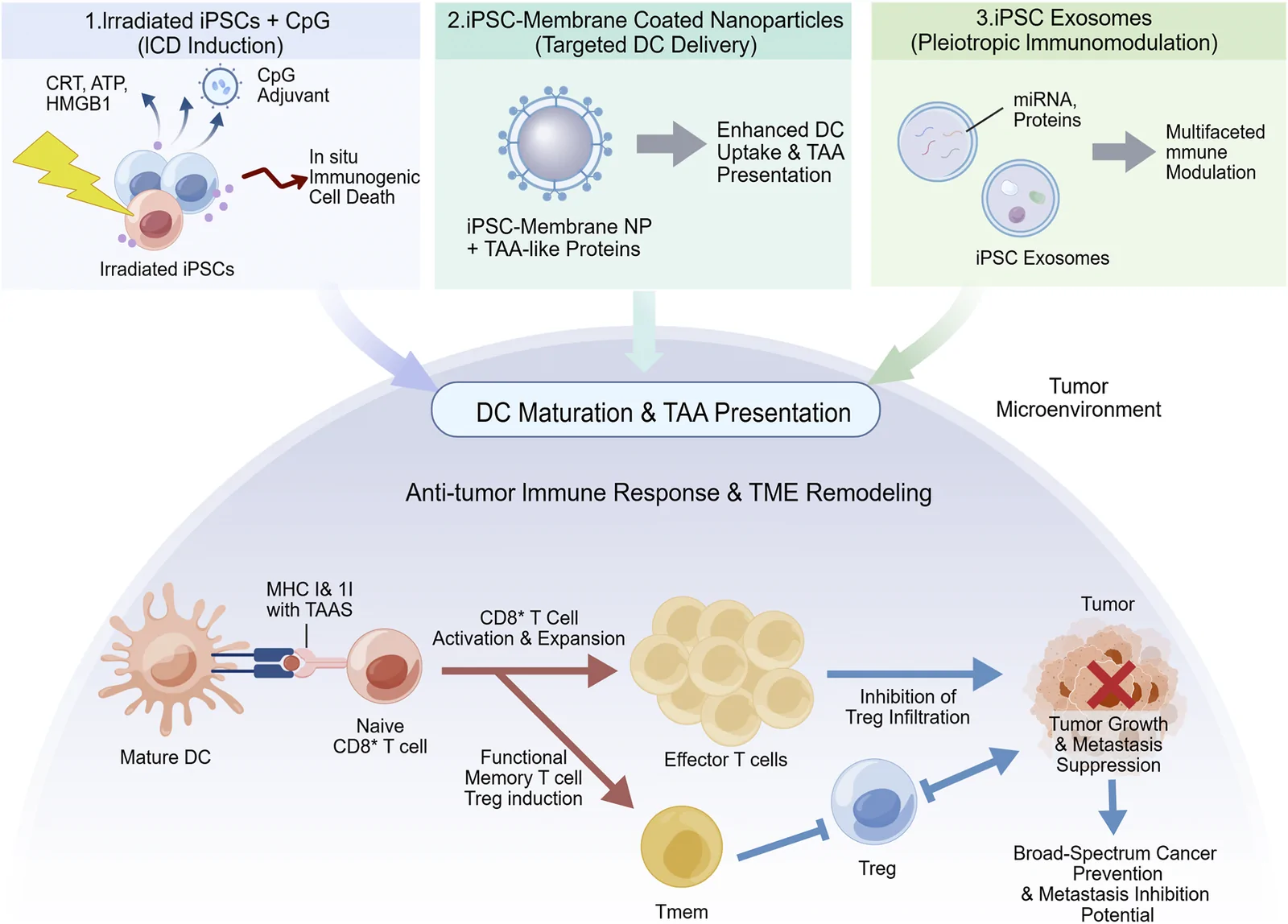
Overview of iPSC-derived tumor vaccines. iPSC-derived tumor vaccines activate antitumor immune responses through three strategies: (1) Irradiated iPSCs combined with CpG adjuvants induce *in situ* immunogenic cell death; (2) iPSC membrane-coated nanoparticles enable targeted delivery to dendritic cells (DCs); (3) iPSC-derived exosomes mediate pleiotropic immunomodulation. These three approaches collectively promote DC maturation and presentation of tumor-associated antigens (TAAs), driving CD8^+^ T cell activation and clonal expansion, inducing functional memory T cell (Tmem) generation, while concomitantly suppressing regulatory T cell (Treg) infiltration.

iPSC-derived exosomes (iPSC-Exos) retain parental tumor-associated antigens but lack the tumorigenic risks of intact iPSCs (Wang et al., 2023; Wang A. Y. L. et al., 2025). Their therapeutic effects mirror direct cell transplantation while significantly reducing safety risks due to their cell-free nature (Wang et al., 2023). The vaccine formed by DCs pulsed with iPSC-Exos (DCs + EXOs) can induce a killing effect in multiple cancer cells and significantly enhance anti-tumor immunity (Wang et al., 2023). iPSC-derived exosome vaccines offer superior cost-effectiveness and therapeutic efficacy compared to other exosome vaccines (Santos and Almeida, 2021), acting by remodeling the TME, enhancing the immune response, and improving antigen delivery. iPSC exosomes carry key antigenic proteins including HNRNPU and NCL, which are important targets for peptide vaccine development (Jwo et al., 2025). Exosome vaccines are more readily taken up by lymphoid organs than irradiated iPSC vaccines, overcoming conventional vaccines’ low delivery efficiency (Wang et al., 2023).

Despite promising preclinical data supporting both prophylactic and therapeutic applications of these tumor vaccines in different syngeneic mouse tumor models, iPSC-derived tumor vaccines have largely remained at the preclinical stage, with no registered clinical trial testing an iPSC-derived tumor vaccine in human patients (Zhai et al., 2024). While other types of iPSC-derived cellular therapies have already been translated into early clinical trials, the translation of iPSC-derived tumor vaccines has been lagging behind, largely due to the tumorigenic potential of residual iPSCs, low immunogenicity, and ineffective delivery of antigens to lymphoid organs (Zhai et al., 2024; Chen et al., 2025). Preclinical and translational evaluation, including the use of humanized mice and patient-derived xenografts, has become essential to predict clinical outcomes and refine the formulations prior to first-in-human trials. At the same time, iPSC-derived tumor organoids may serve as useful tools for evaluating the performance of tumor vaccines (Zhai et al., 2024). Further safety profiling, standardized production processes, and use of advanced delivery systems are among the crucial steps required to translate iPSC-derived tumor vaccines into clinical practice (Chen et al., 2025). Importantly, the antitumor efficacy of iPSC-based tumor vaccines has so far been demonstrated almost exclusively in syngeneic mouse models; caution should be exercised when extrapolating these preclinical findings to human patients as several proposed mechanisms of action (e.g., exosome-mediated immune modulation) remain incompletely characterized.

## 4 Malignant transformation of iPSCs and pluripotency reprogramming of cancer cells

While the previous sections discussed how iPSC-derived immune cells and tumor vaccines can be applied in the treatment of various diseases, this section covers how iPSCs can serve as an experimental model to study cancer, in particular, CSCs. CSCs and iPSCs share several similarities, such as the ability to self-renew, the ability to differentiate into many cell types, and the requirement for pluripotent transcriptional programs. Therefore, iPSCs can serve as a unique *in vitro* platform for studying the cellular and molecular mechanisms of malignant transformation and tumorigenesis (Sarker et al., 2024). There are two experimental approaches to studying cancer with iPSCs. First, the malignant transformation of iPSCs into CSC-like cells enables scientists to observe the process of oncogenesis and to identify the genetic, microenvironmental, and extracellular vesicle signaling mechanisms that trigger cellular stemness and malignancy. Second, reprogramming cancer cells into iPSCs resets them to a pluripotent state while retaining their tumorigenic memory, thereby providing a source of patient-specific CSCs. It is important to highlight that although the mechanistic findings gained through these iPSC models have been primarily achieved through *in vitro* culture experiments and xenotransplantation in immunocompromised mice, it is not yet known to what extent these findings represent human tumor formation *in vivo*.

### 4.1 CSC-like cells derived from iPSCs

#### 4.1.1 CSCs

CSCs possess both dual self-renewal and differentiation potential, forming hierarchical tumor structures that drive tumorigenesis, progression, metastasis, and resistance (Yu et al., 2012; Zhang et al., 2024). They are key players in tumor recurrence and drug resistance as they are difficult to eradicate using conventional chemotherapy and targeted therapy because of the presence of drug efflux pumps and anti-apoptotic pathways, and interactions with the TME (Safa, 2020; Zhang et al., 2024). These attributes make CSCs a core factor in determining drug tolerance, disease recurrence, and distant metastasis during tumor treatment.

#### 4.1.2 Genetic manipulation-mediated transformation of normal iPSCs into CSC-like cells

iPSCs and CSCs are significantly similar at the molecular level, offering a novel model to decipher tumor initiation and progression.Specific genetic manipulations can transform iPSCs into cells with CSC properties (Sarker et al., 2024; Figure 3). Because certain oncogenic mutations can be lethal to iPSCs, inducible expression systems precisely regulate these mutations and CSC marker expression to obtain CSC-like progenitor cells. Since the cellular origin of cancer may differ from metastasis-driving CSCs, iPSCs harboring oncogenic mutations may better model cancer initiation than traditional CSC models (Visvader, 2011; Shamsian et al., 2022). In brain tumor modeling, specific oncogenic mutations remodel iPSC stemness networks. Inducible endogenous H3.3-K27M mutation enhances proliferative capacity and stemness in iPSC-derived NSCs, leading to glioma-like cell formation (Haag et al., 2021). This mutation locks cells in an immature state by disrupting PRC2 complex function and enhancer activity, triggering tumorigenesis (Brien et al., 2021). Liu et al. used mid-trimester human brain neural stem cells/glial progenitor cells/oligodendrocyte progenitor cells (NSCs/GPCs/OPCs) as a homologous starting point and jointly introduced driver TP53 R^17^5H/shNF1, CDK4/EGFR/PDGFRA, and other driver mutations. After transplantation *in vivo*, the cells rapidly generated mouse tumors that reproduced the heterogeneity of human gliomas, showing the typical expression of CSC markers and tumor initiation ability (Liu et al., 2025).

**FIGURE 3:**
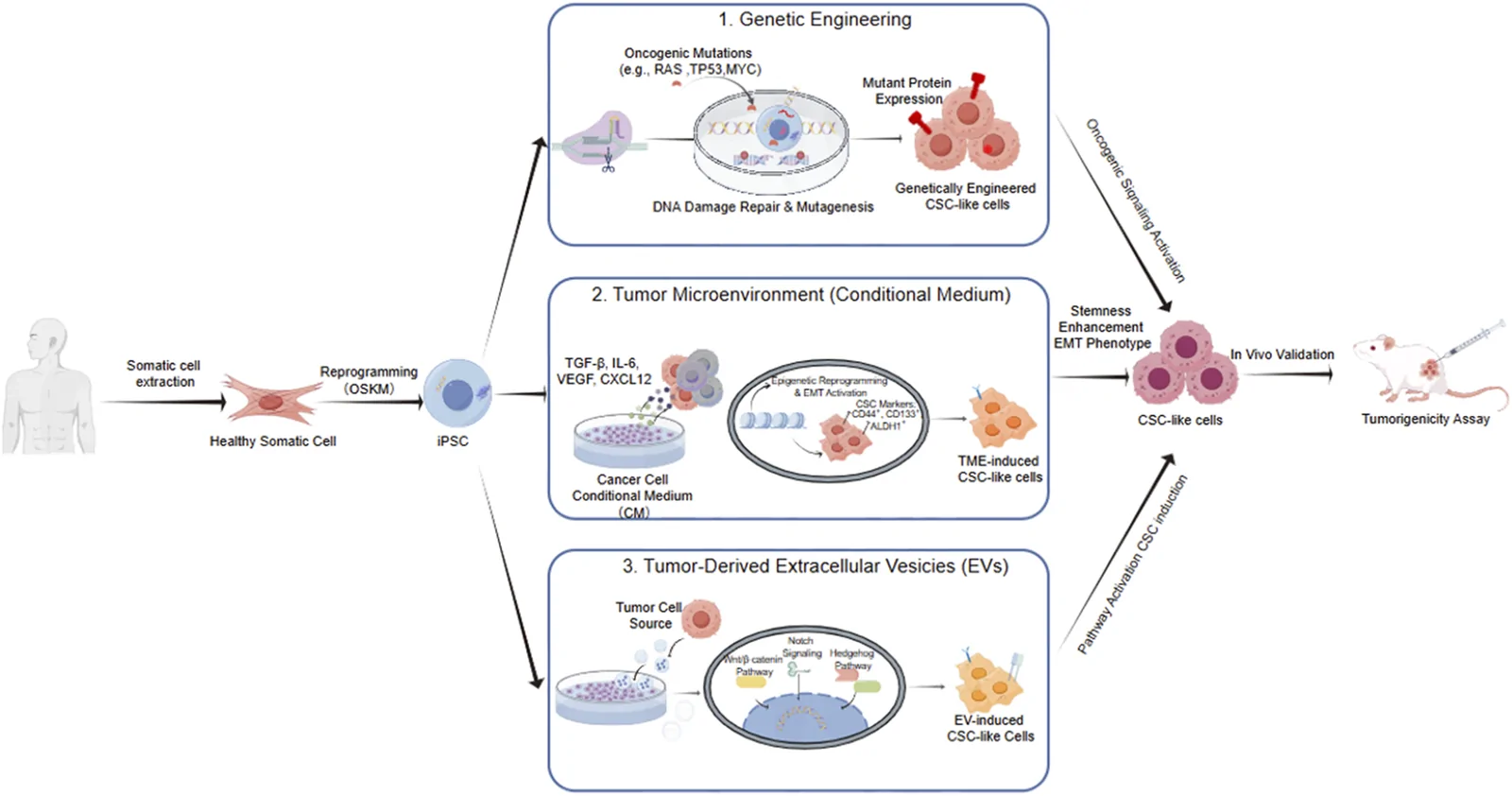
Cancer stem cell-like cells derived from iPSCs. Three approaches for generating iPSC-derived CSC models: first, genetic engineering of normal iPSCs to promote the derivation of cancer stem cell-like cells; second, culture of iPSCs in cancer cell-conditioned medium, in which exposure to tumor microenvironmental factors drive distinct CSC characteristics and *in vivo* tumorigenic capacity; third, delivery of specific molecules via tumor-derived extracellular vesicles to promote iPSC-to-CSC conversion.

The core mechanism underlying the transformation of gene-edited iPSCs into CSC-like cells involves coordinated perturbation of key signaling pathways. Loss of TP53 has been shown to significantly expand the CSC population and promote tumor growth, whereas activation of p53 inhibits the self-renewal ability of CSCs (Rodriguez-Ramirez et al., 2022). PTEN deficiency leads to reprogramming of cells into a more stem-like phenotype by affecting PAX2 expression and simultaneously enriching the subpopulation with acquired chemotherapy resistance (Russo et al., 2021; Yanushko et al., 2025). This transformation exhibits significant tissue specificity: within the neural lineage, the H3.3-K27M mutation is lethal to iPSCs but promotes proliferation in neural stem cells (Haag et al., 2021), while the critical role of PTEN affects the directional migration and differentiation homeostasis of neural stem cells (Li Y. et al., 2024), further explaining why specific mutation combinations can efficiently induce the characteristics of CSCs in glioma models (DeSouza et al., 2025; Jung and Benvenisty, 2025).

KRAS p. G12V is a common oncogenic mutation introduced into iPSCs to model cancer initiation. RAS mutations exhibit specific tropism across cancer types, and highly active oncogenic RAS signaling causes oncogenic stress (Li and Counter, 2021). Busley et al. (2023) established an iPSC line carrying the KRAS p.G12V mutation, resulting in constitutive RAS-MAPK pathway activation that drives proliferation, metabolic reprogramming, and malignant transformation. Thus, KRAS-mutant iPSCs resemble cancer cells morphologically and simulate TME effects molecularly. These findings verify the reliability of the iPSC-CSC transformation model and reveal differential effects of oncogenic mutations across developmental stages, providing crucial evidence for conditional gene regulation driving CSC generation.

#### 4.1.3 The TME promotes the transformation of iPSCs into CSC-like cells

Current research widely supports the “microenvironmental education” hypothesis, wherein the TME drives phenotypic shifts in normal stem cells (Booth et al., 2011; Marotta and Polyak, 2009; Ajani et al., 2015; Figure 3). As the master regulatory niche for tumor initiation and progression, the TME features cancer cells as dominant agents driving tumorigenesis. Diverse stromal cells interact with the extracellular matrix and other factors to jointly promote tumor growth and maintenance (Osman et al., 2020). This process can be modeled by exposing iPSCs to cancer cell-conditioned medium or through direct physical interactions with tumor cells to trigger stemness reprogramming (Afify et al., 2020; Chen et al., 2012). Ling et al. examined both the direct co-culture of miPSCs with mouse Lewis lung cancer cells (LLC) and exposure of miPSCs to cancer cell conditioned medium (CM). CSCs derived by exposing miPSCs to the cancer cell CM were more successful. Although direct co-culture enabled cell contact and dual paracrine stimulation, miPSCs exhibited poor adhesion, proliferation arrest, and massive cell death after the second passage, preventing adequate cell yield for spheroid formation or tumorigenicity assays, with no observable CSC phenotypes. Conversely, exposure to purified CM eliminated contact-dependent cytotoxicity while enriching key malignant transformation factors. Resulting cells displayed CSC-like properties, including self-renewal in sphere formation and efficient tumorigenesis in nude mice. Soluble factors, not cell-cell contact, are thus essential for transformation (Chen et al., 2012; Calle et al., 2016; Afify et al., 2020; Nair et al., 2017; Prieto-Vila et al., 2016).

In recent years, the experimental strategy of exposing iPSCs to cancer cell CM has become one of the most concise, efficient, and reproducible modeling methods in CSC research (Afify et al., 2019; Chen et al., 2012). The core mechanism involves the continuous secretion of large amounts of soluble factors, including growth factors, exosomes, metabolic intermediates, and active fragments after proteolytic cleavage during *in vitro* proliferation of cancer cells (Osman et al., 2020). This culture medium precisely recreates the biochemical characteristics of the *in-situ* microenvironment in both spatial and temporal dimensions by reconfiguring the paracrine-endocrine signaling network within the TME. This effectively avoids contact toxicity or competitive proliferation interference between intact cancer cells and iPSCs. Collectively, these advantages render CM-based induction a standardized, cross-tumor modeling paradigm.

As shown in Table 1, Nair et al. (2017) induced CSC-like cells by treating miPSCs with breast cancer cell CM. These cells maintained tumorigenicity upon serial transplantation in nude mice and retained CSC characteristics. Their differentiated cancer-associated fibroblast-like cells exhibited high invasiveness and upregulated fibroblast markers. Afify et al. (2023) used MDA-MB-231 CM to establish a tumorigenic CSC model (miPS-MB231 cm). When implanted into mouse mammary fat pads, these cells showed high host tissue compatibility. Despite the downregulation of some stemness markers, core CSC characteristics persisted. Cancer-associated fibroblast-like cells showed high invasiveness and upregulated fibroblast marker expression. Afify et al. (2020) stimulated miPSCs with hepatocellular carcinoma Huh7 CM, generating liver CSCs (miPS-Huh7cm) within 4 weeks without genetic manipulation. After orthotopic transplantation into the liver, 100% of these cells formed highly proliferative and invasive malignant tumors and maintained tumorigenicity for three consecutive generations. RNA sequencing revealed TME-driven CSC self-renewal and metastasis via phosphatidylinositol 3-kinase-protein kinase B signaling pathway (PI3K-AKT), vascular endothelial growth factor (VEGF), and epithelial-mesenchymal transition (EMT) pathways (involving MMP2/9, Slug). Du et al. (2020) found that miPSCs cultured in LLC CM combined with a GSK-3β inhibitor (CHIR99021), MEK inhibitor (PD0325901), or Src/Abl/c-Kit inhibitor (Dasatinib) could be efficiently converted to CSCs within 1 week. The resulting CSCs showed enhanced tumorigenicity. Inhibitors activated PI3K-AKT by blocking ERK1/2 phosphorylation and GSK-3β activity, promoting stemness factor upregulation and sphere formation. Exposing hiPSCs to BxPC3 pancreatic cancer CM with AZD6244 (MEK inhibitor) and CHIR99021 induced malignant CSCs (hiPS-Bxcm) within 4 weeks. These cells exhibit the typical CSC marker, can form spheroids under low-adhesion conditions, and generate invasive tumors after pancreatic orthotopic transplantation. Despite variations in cancer type and species origin, these studies collectively demonstrate that cancer cell CM consistently confers core CSC hallmarks including self-renewal, tumorigenicity, and metastatic potential via convergent PI3K-AKT, EMT, and angiogenesis pathway activation, underscoring a conserved TME-driven reprogramming mechanism across malignancies.

**TABLE 1:** *In vitro* conversion of iPSCs into CSC-like cells via tumor microenvironment induction.

iPSC type	Inducing microenvironment sources (cancer cells)	Core transformation strategy	Signal inhibitors/drugs used	Phenotypic diversity and specific markers	Activated/inhibited signaling pathways	*In vivo* tumorigenicity and metastatic potential	Ref.
miPSC	Mouse Lewis lung cancer cells (LLC)	CM induction (LLC-CM, 1:1, 4 weeks); subcutaneous xenograft	None (pure CM)	GFP^+^/Nanog^+^; upregulated Oct3/4, Sox2, Klf4, c-Myc; enhanced self-renewal; sphere formation	PI3K/AKT activation	Tumors at 3 weeks (5 × 10^5^ cells); central necrosis with peripheral proliferation	Chen et al. (2012)
	Human breast cancer cells (MDA-MB-231)	CM induction (MDA-MB-231-CM, 1:1, 4 weeks); xenotransplantation	None (pure CM)	CD44^+^/CD133^+^/EpCAM^+^; downregulated Oct3/4/Sox2/Nanog; retained CSC traits	PI3K/AKT,MAPK, ER signaling	Invasive mammary tumors; local infiltration and vascular invasion; GFP^+^ CSCs along ductal basement membranes	Afify et al. (2023)
	Human hepatocellular carcinoma cells (Huh7)	CM induction (Huh7-CM, 50%, 4 weeks); orthotopic hepatic transplantation	None (pure CM)	CD44^+^/CD24^+^/CD133^+^, upregulated Nanog/Oct3/4/Sox2/Klf4; sphere formation; HCC markers (GPC3, CK19)	PI3K/AKT, MAPK, Ras, Wnt, inflammatory signaling	100% tumor incidence, large invasive tumors; pulmonary metastasis (tail vein)	Afify et al. (2020)
	Human breast cancer cell (T47D,BT549)	CM induction (T47D/BT549-CM)	None (pure CM)	GFP^+^; upregulated CD44/CD133; sphere formation; CAF differentiation (invasive CAFLCs with CAF markers)	TGF-β/IL-6 autocrine activation	Subcutaneous tumorigenicity; sustained upon serial transplantation	Nair et al. (2017)
	Mouse Lewis lung carcinoma cells (LLC)	LLC-CM + single inhibitor (1 week; CM alone: 4 weeks)	MEK inhibitor (PD0325901); GSK-3β inhibitor (CHIR99021); Src/Abl/c-Kit inhibitor (Dasatinib)	Upregulated Nanog/Oct3/4/Sox2/Klf4; enhanced sphere formation; vascular endothelial differentiation; GFP^+^ (60%–80%)	PI3K/AKT activation; ERK1/2 inhibition	100% tumor incidence at 6 weeks (1 × 10^6^ cells, s.c.); histologically confirmed malignancy	Du et al. (2020)
hiPSC	Pancreatic cancer cells (BxPC-3)	BxPC-3-CM + AZD6244+CHIR99021	MEK inhibitor (AZD6244); GSK-3α/β inhibitor (CHIR99021)	CD44^+^/CD24^+^/CD133^+^/EpCAM^+^; sphere formation; invasive orthotopic pancreatic tumors; elevated p-AKT, suppressed p-ERK1/2	PI3K/AKT activation; MEK/ERK inhibition	Invasive pancreatic tumors; stable tumorigenicity upon serial transplantation	Hassan et al. (2023)

ER signaling: estrogen receptor signaling pathway; s.c.: subcutaneous injection.

#### 4.1.4 EVs promote the transformation of iPSCs into CSC-like cells

The EVs secreted by cancer cells are highly specialized intercellular communication mediators in the TME that mediate complex biological processes in a paracrine manner. TDEVs can reprogram iPSCs into CSC-like cells with tumorigenic properties by delivering specific molecular cargos (Su et al., 2021; Wang, 2022; Semeradtova et al., 2025) (Figure 3). This process redefines the phenotype of iPSCs and provides cells with the capacity for tumorigenesis and metastasis. Extracellular vesicle uptake occurs through multiple pathways including plasma membrane fusion and various endocytic mechanisms such as clathrin-mediated endocytosis and caveolin-dependent endocytosis enabling cargo delivery that modulates intracellular signaling. TDEV molecules play a crucial role in intercellular communication by regulating key signaling pathways and epigenetic mechanisms, thereby driving the phenotypic transformation of iPSCs. Transformed CSC-like cells exhibit typical characteristics such as enhanced self-renewal ability, treatment resistance, and metastatic potential. These characteristics are sustained by core pluripotency transcription factors, including OCT4/SOX2 (Takahashi and Yamanaka, 2006; Boyer et al., 2005), or may lead to chemotherapy resistance through the delivery of drug resistance-related proteins by EVs (Tang et al., 2022; Yang et al., 2022). Reprogramming efficiency depends on EV subtype heterogeneity, cargo composition, and the differentiation state of recipient cells. The reprogramming effect of TDEVs on iPSCs depends on the specific molecular combination carried by the EVs and the microenvironmental response of the target cells. This mechanism provides a new model for studying the origin of CSCs and developing targeted therapies (Ruivo et al., 2022).

Each of the above strategies is unique in its merits and is tailored to specific research goals. TME-based induction (including CM) most faithfully recapitulates the generation of patient-derived CSCs by driving non-genetic phenotypic reprogramming through the complete paracrine-endocrine signaling network, closely mirroring microenvironmental education *in vivo*. In contrast, genetic manipulation provides the highest reproducibility and genetic homogeneity, making it ideal for high-throughput drug screening and precise target validation. Among microenvironment-mimicking approaches, CM exposure best preserves tumor microenvironment fidelity by encompassing soluble factors, exosomes, and metabolic intermediates that reconstruct the biochemical complexity of the native niche across spatial and temporal dimensions. Although tumor-derived EVs alone can efficiently transfer oncogenic cargo and induce CSC-like properties, they represent a simplified subset of TME signals; thus, they are better suited for mechanistic dissection of intercellular communication than for holistic modeling of tumor initiation. Ultimately, the choice of approach should be guided by the research priorities: modeling physiological tumor-microenvironment crosstalk, dissecting specific molecular drivers, or establishing scalable therapeutic screening pipelines.

### 4.2 Reprogramming to generate cancer-derived iPSCs

Cancer cell reprogramming has become a cutting-edge technology in cancer biology. Using iPSC technology, researchers can reprogram differentiated cancer cells into iPSCs that are similar to CSCs, termed cancer-derived iPSCs (Shamsian et al., 2022).

#### 4.2.1 Similarities and reprogramming between cancer cells and iPSCs

Traditionally, CSCs are thought to originate from the malignant transformation of normal stem cells. However, differentiated mature cancer cells can also gain stem cell properties through dedifferentiation or reprogramming, which is closely associated with tumor progression and drug resistance (Saito et al., 2019). Since 2010, accumulating evidence has demonstrated that cancer cells, with their inherent self-renewal capacity, telomerase activity, and epigenetic plasticity, can bypass certain reprogramming barriers and even reverse malignant phenotypes upon the introduction of exogenous reprogramming factors. Thus, cancer cells may serve as a novel source of iPSCs (Miyoshi et al., 2010).

#### 4.2.2 Principal sources for generating cancer-derived iPSCs

The initial cell material that generates cancer-derived iPSCs is crucial as it directly determines the representativeness, stability, and scope of application of the final model. According to existing literature, these sources can be divided into two main categories: established cancer cell lines and primary tumor samples.

Cancer cell lines are a group of cancer cells cultured *in vitro*. Many early cancer-iPSC studies have mostly used mature cancer cell lines, as shown in Figure 4. Researchers have successfully generated iPSCs from a variety of human cancer cell lines, including malignant tumors of the blood (Curry et al., 2015), digestive system tumors (Franco et al., 2016), and other solid tumors (Chun et al., 2010). CSCs are a subgroup of cancer cells that exhibit stem cell-like characteristics and are important sources of cancer iPSCs. Cancer cell lines can be obtained and cultured easily. They are usually obtained from commercial cell banks (e.g., American Type Culture Collection (ATCC)). The culture conditions are standardized and can be easily replicated in the laboratory, providing sufficient starting materials for the reprogramming experiments. The genome and transcriptome of the most commonly used cancer cell lines have been sequenced, and their key mutations and molecular characteristics are known. This provides a clear reference for subsequent analysis of the genetic fidelity of cancer-derived iPSCs. In addition, the results obtained using cancer cell lines are highly reproducible because of high homogeneity; the results obtained from experiments using the same cell lines and reprogramming schemes in different laboratories are comparable and reproducible, which facilitates the validation of research findings.

**FIGURE 4:**
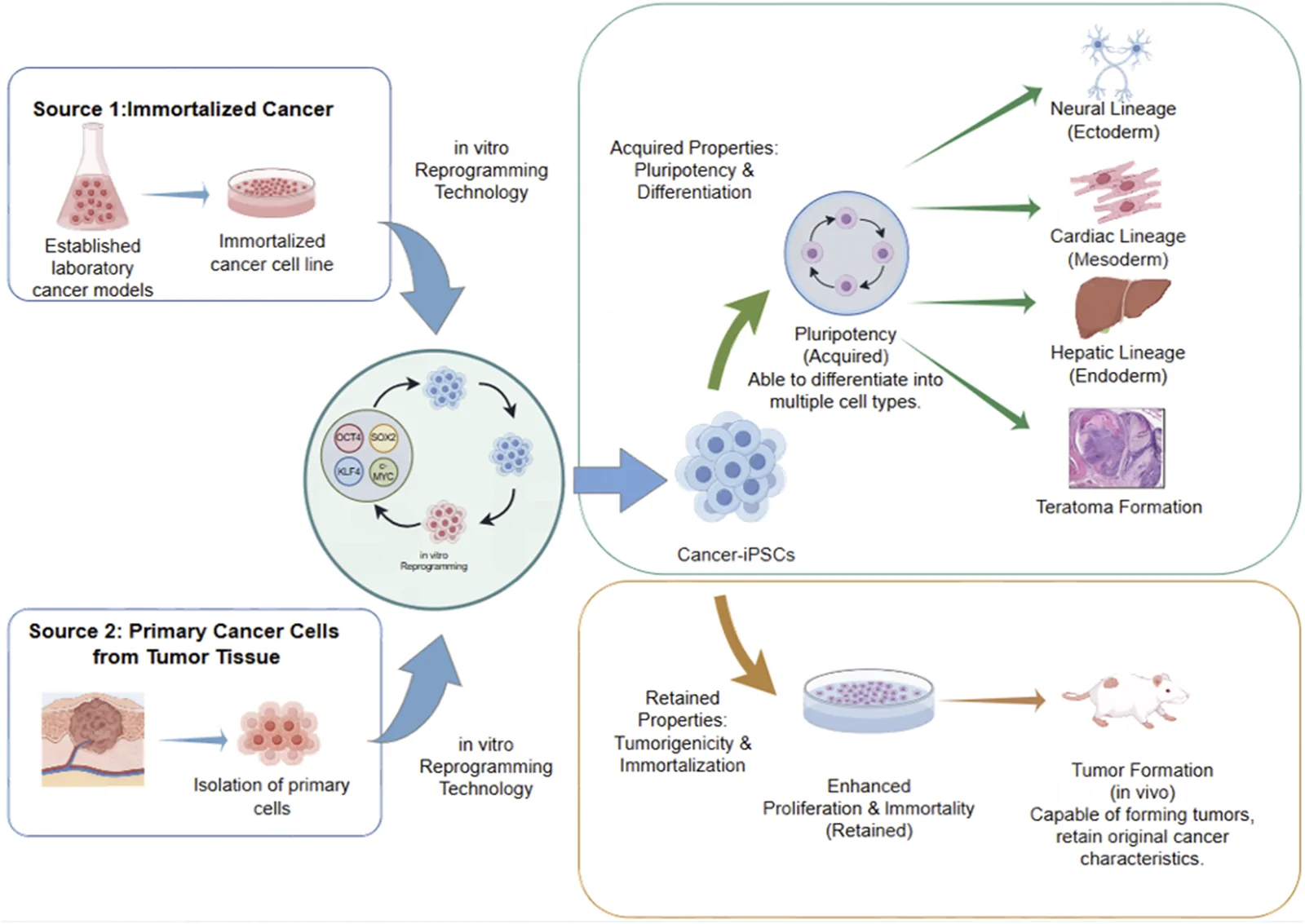
Two approaches for cancer-derived iPSC generation. Cancer cells isolated from tumor tissue blocks can be reprogrammed *in vitro* to generate cancer-derived iPSCs. Alternatively, cancer-derived iPSCs can be derived from induced immortalized cancer cell lines. These cells exhibit immortalization and proliferative capacity, retain tumorigenic potential, and acquire pluripotency and teratoma-forming capability during the reprogramming process.

However, the use of cancer cell lines has certain limitations. After long-term *in vitro* culture, selective pressure and genomic drift occur in the cell lines, and many key characteristics of the original tumor may be lost, especially heterogeneity inside the tumor (Kim and Zaret, 2015). As the genetic background may differ from that of primary tumors, the accuracy of a cancer-derived iPSC model based on cell line sources in predicting the true response of patients to drugs may be limited. Changes in epigenetic states also have significant limitations. Long-term two-dimensional culture environments profoundly change the epigenetic landscape of cells, because of which their biological behavior may differ from that of cancer cells in the body, which affects the reprogramming process itself (Franco et al., 2016).

To overcome cancer cell line limitations, researchers isolate cells directly from patient tumor tissues or biopsies to generate cancer-derived iPSCs for clinically relevant models (Figure 4). Currently, cells can be successfully isolated and reprogrammed from various primary tumor samples (Wan et al., 2015), such as cancer cells isolated from solid tumor tissues and leukemia cells isolated from the peripheral blood or bone marrow of patients with hematological tumors. Moreover, Ahn et al. generated iPSCs from tumor tissues of patients with combined hepatocellular carcinoma and cholangiocarcinoma to investigate the pathogenesis of this rare liver cancer (Ahn et al., 2022).

The main advantage of using primary tumor samples is their high clinical relevance. These cells originate directly from the patient’s tumor tissue and preserve the genetic mutation spectrum, chromosomal abnormalities, and heterogeneity of the original tumor to the greatest extent (Papapetrou, 2019). The models based on them are ideal for personalized drug screening and biomarker research. Moreover, different cell subgroups can be isolated from primary samples to construct specific cancer-derived iPSC models for distinct intratumoral clones, which is critical for investigating tumor evolution and drug resistance mechanisms. Additionally, freshly isolated cancer cells better reflect *in vivo* epigenetics, facilitating the study of gene expression regulation and cell plasticity.

The use of primary tumor samples is challenging. Such samples are difficult to acquire, with limited quantities typically obtained through surgical resection or biopsy, thereby hindering large-scale and repeated experiments. Isolation and culture of primary tumor cells demands highly efficient separation, purification, and expansion from complex tumor tissues, posing technical difficulties. Furthermore, marked heterogeneity in cellular composition, viability, and genetic background across individual patients compromises experimental reproducibility and complicates standardization.

#### 4.2.3 Reprogramming strategies and research advancements in cancer-derived iPSC generation

Cancer-derived iPSC generation is a complex multistep process that draws on standard iPSC generation technology and is adjusted to the specificity of cancer cells. Introducing reprogramming factors is essential for this process. The choice of delivery method affects reprogramming efficiency, genomic safety, and subsequent applications. Methods are categorized as integrated or non-integrated. Retroviral or lentiviral vectors are primary integrating vectors; these randomly integrate Yamanaka factor sequences into the host genome for long-term stable expression (Kotini et al., 2015). Integrated vectors offer high, optimized reprogramming efficiency but carry the risk of insertion mutations. Exogenous gene integration may inactivate key endogenous genes or activate nearby oncogenes. The introduction of new genetic mutations from non-primitive tumor sources will interfere with cancer research and analysis. Non-integrating vectors can be used to avoid the risk of integration. For cancer-iPSC research, the non-integration method is more suitable to ensure that the genetic variation in the generated iPSC model originates entirely from the original cancer cells and is not introduced by the reprogramming technology itself. For example, the Sendai virus, an RNA virus that replicates in the cytoplasm and does not integrate into the host DNA, has been used for delivery (Lee et al., 2015). After reprogramming, viruses can be easily removed using temperature-sensitive mutations or simple passaging, which is currently one of the most commonly used and efficient non-integration methods. Delivery using episomal plasmids is another preferred way (Moore et al., 2015). These plasmids carry reprogramming factors and special replication elements such that they can replicate autonomously in the nucleus, and their number reduces with each cell division. This method is moderately efficient but completely virus-free.

Considering the impact of delivery strategy on model fidelity, cancer-iPSC technology can be valuable in research on the mechanisms of cancer development and treatment design. Reprogramming cancer cells into a multipotent state can normalize the malignant phenotypes of tumor cells, which enables the development of new treatment strategies. However, cancer-derived iPSCs are mainly used as a research model rather than a direct therapeutic. This type of research lays the foundation for analyzing the mechanisms of drug resistance in tumors and formulating strategies to prevent recurrence. Table 2 summarizes current research on various malignant solid tumor cells reprogrammed into a pluripotent state.

**TABLE 2:** Overview of research on cancer cell reprogramming.

Species	Cancer cell type	Reprogramming methods	Pluripotency marker expression	Teratomaformation	Tumorigenicity	Drug sensitivity	Differentiation products	Ref
Human	Human melanoma Cells (A375, MDA-MB-435)	Sendai virus OSKM	Increased	N/A	N/A	N/A	Three germ layers	Taheri et al. (2019)
	Pancreatic ductal adenocarcinoma (PDAC-247)	Episomal vectors OSKM	Increased	N/A	Decreased	N/A	N/A	Khoshchehreh et al. (2019)
	Prostate cancer cells (22RV1)	Sendai virus OSKM	N/D	N/A	N/A	N/A	Three germ layers	Zhang et al. (2020)
	RDEB-cSCCs	Episomal vectors OSKM	Increased	N/A	Decreased	N/A	Keratinocytes	Rami et al. (2020)
	Human sarcoma cells (HOS, SAOS2, MG63, SW872, SKNEP)	Lentivirus OSKMLN	Increased	No	Decreased	N/A	Endoderm and ectoderm, mature connective tissue and red blood cells	Zhang et al. (2013)
	Lung adenocarcinoma cells (A549)	Lentivirus OSLN hypoxia (HIF)	N/D	No	Decreased	N/A	N/A	Mathieu et al. (2011)
	ovarian can Cer cell (PEO4)	Retroviruses OSKM/OSKG	Increased	N/A	N/A	More resistant to cisplatin and taxol	Three germ layers	Bindhya et al. (2021)
Mouse	Melanoma cells (R545)	Lentivirus OKM	Increased	Yes	Decreased	N/A	N/A	Utikal et al. (2009)
	ESCs derived germ cell tumors	ESC medium	Increased	Yes	Decreased	N/A	Embryonic and extraembryonic lineage	Taguchi et al. (2021)
	Glioblastoma multiform cells	Retroviral plasmids OSK + PD98059	Increased	N/A	N/A	N/A	N/A	Vatanmakanian et al. (2019)

N/A: not applicable; N/A: not determined; OKM: OCT4, KLF4, c-MYC; OSKM: OCT4, SOX2, KLF4, c-MYC; OSKMLN: OCT4, SOX2, KLF4, c-MYC, LIN28, NANOG; OSLN: OCT4, SOX2, LIN28, NANOG; OSKG: OCT4, SOX2, KLF4, GLIS1; OSK: OCT4, SOX2, KLF4; RDEB-cSCCs: Recessive dystrophic epidermolysis bullosa (RDEB)-derived cutaneous squamous cell carcinomas (cSCCs).

## 5 Discussion and conclusions

By providing infinitely amplified and genetically editable homologous cells, iPSC technology has helped overcome bottlenecks such as donor scarcity and batch-to-batch variability in traditional cell therapies. iPSC technology has opened new avenues in cancer treatment and has demonstrated potential in three major areas: immune cell therapy, tumor vaccines, and CSC research. These three application areas offer distinct advantages. iPSCs can be used to differentiate into immune cells and rare immune cell types, which expands the diversity of clinical treatment options (Basilio-Queiros et al., 2025). iPSC-derived tumor vaccines can express individual-specific tumor antigens on autologous cells, enabling highly personalized treatment. iPSC-derived CSC-like cells and cancer-derived iPSCs are highly similar to native CSCs, which can effectively solve the problem with obtaining, culturing, and amplifying clinical samples. In addition, these approaches have synergistic potential. The key targets screened by the model can optimize the vaccine antigen design (Zhai et al., 2024; Huang et al., 2024). The immune response data induced by vaccines can be used to back-feed the model to verify the immune-microenvironment interaction. The tumor heterogeneity revealed by the model can guide the targeted modification strategy of iPSC-derived immune cells (Bernardes de Jesus et al., 2020).

Despite these promising advances, the limitations and disadvantages of iPSC-guided therapies must be frankly acknowledged, as they currently constrain the clinical and translational impact of this field. First, tumorigenicity remains the most fundamental safety concern: reprogramming factors and residual undifferentiated iPSCs carry carcinogenic potential and point-mutation risk (Zhong et al., 2022; Qiao et al., 2020; Fang et al., 2024). Notably, even “unexpected proliferative off-target cells” have been detected after transplantation of iPSC-derived products in preclinical models, implying that current purity requirements might be inadequate (Hiyoshi et al., 2024). Second, immunogenicity is more complex than initially anticipated: abnormal gene expression and residual pluripotent cells can elicit unexpected immune responses even in syngeneic settings, and allogeneic products require either HLA matching or hypoimmunogenic engineering, both of which add manufacturing burden. Third, most iPSC-T cell therapies remain in phase I dose-escalation trials; iPSC-derived tumor vaccines have not yet entered any registered clinical trial; and iPSC-Macs are still awaiting first-in-human validation (Fang et al., 2025; Zhai et al., 2024; Reiss et al., 2025). This slow clinical translation reflects persistent technical bottlenecks, including low and variable differentiation efficiency, insufficient functional maturation of iPSC-derived effector cells, limited *in vivo* persistence, and batch-to-batch heterogeneity that complicates standardization. Fourth, economic and regulatory barriers are substantial: the absence of standardized biodistribution and tumorigenicity assessment protocols has led to inconsistent results that hinder regulatory approval (Nakayama et al., 2025), while high production costs and the lack of unified potency assays impede large-scale clinical application. Finally, although iPSC technology avoids embryonic stem cell controversies, patient data privacy, intellectual-property ownership in commercialization, and conflicts of interest still require proper regulation. These limitations collectively explain why iPSC-guided therapies have not yet achieved their anticipated clinical impact, and they define clear priorities for further research.

In order to realize the large-scale supply of ready-made cells, it is necessary to develop general-purpose HLA engineering products or immunocompatible designs. This would reduce the matching needs of patients and improve anti-tumor activity, lasting efficacy and safety. In the future, the development of iPSC-derived tumor vaccines should focus on engineering transformation, combination strategies, and the design of innovative delivery systems. Introducing a neoantigen engineering strategy into iPSC technology and developing a neoantigen-iPSC vaccine can induce a stronger antigen-specific T cell response and significantly improve the anti-tumor effect. Nanotechnology can be used to overcome delivery obstacles and improve targeting. Furthermore, radiotherapy can alter the TME and promote activation of DCs. Intratumoral injection of iPSC-DCs or systemic application of the NA-iPSC vaccine can significantly enhance immune response to anti-tumor agents. Therefore, combining iPSC-derived tumor vaccines with other immunotherapies, chemotherapy, or photodynamic therapy is also a future direction. Given that no iPSC-derived tumor vaccine has yet entered clinical trials, rigorous preclinical evaluation in humanized mouse models and patient-derived xenografts, together with standardized manufacturing protocols and advanced delivery systems, will be critical to advance iPSC-based tumor vaccines from bench to bedside (Zhai et al., 2024).

The CSC model based on iPSCs can simulate the TME, which helps analyze the carcinogenic process and develop small-molecule drugs targeting CSCs (Liu et al., 2023; Lee et al., 2021). Developing a fully chemical small-molecule reprogramming method would avoid gene insertion, thereby improving the safety and efficiency of the reprogramming process (Cerneckis et al., 2024). Single-cell RNA sequencing has been applied to deeply analyze the heterogeneity of reprogramming trajectories and CSCs, enabling the identification of key transcription factors that enhance reprogramming efficiency (He et al., 2023). Additionally, 3D cultures based on iPSCs can better simulate the *in vivo* TME; compared with traditional 2D cultures, 3D models more realistically replicate immune cell infiltration and the immunosuppressive microenvironment (Si et al., 2025). iPSC technology offers a bidirectional strategy for CSC research; it facilitates carcinogenesis mechanism analysis using normal cell-derived CSC models and enables cancer cell reprogramming into cancer-derived iPSCs for disease modeling. Future efforts should focus on improving reprogramming efficiency, establishing standardized systems, mitigating tumorigenic risks, and accelerating clinical translation to unlock iPSC potential in cancer treatment and regenerative medicine, thereby providing novel insights for CSC-targeted therapeutic development.

iPSC technology is reshaping cancer research. Its multidimensional applications in cell therapy, vaccine development, and disease modeling offer innovative solutions to address the global cancer burden. With in-depth insights into the biological commonalities between iPSCs and cancer cells, coupled with advances in bioengineering technology, iPSCs are expected to become core players bridging basic research and clinical translation, thereby advancing oncology into a new era of precision medicine.
